# Behavioral changes and gene profile alterations after chronic 1,950‐MHz radiofrequency exposure: An observation in C57BL/6 mice

**DOI:** 10.1002/brb3.1815

**Published:** 2020-08-28

**Authors:** Ye Ji Jeong, Yeonghoon Son, Hyung‐Do Choi, Nam Kim, Yun‐Sil Lee, Young‐Gyu Ko, Hae‐June Lee

**Affiliations:** ^1^ Division of Basic Radiation Bioscience Korea Institute of Radiological & Medical Sciences Seoul Korea; ^2^ Division of Life Sciences Korea University Seoul Korea; ^3^ Primate Resources Center Korea Research Institute of Bioscience and Biotechnology (KRIBB) Jeonbuk Korea; ^4^ Department of EMF Research Team Radio and Broadcasting Technology Laboratory ETRI Daejon Korea; ^5^ School of Electrical and Computer Engineering Chungbuk National University Cheongju Korea; ^6^ Graduate School of Pharmaceutical Sciences Ewha Womans University Seoul Korea

**Keywords:** behavioral alteration, gene profiling, memory, neurogenesis, RF‐EMF

## Abstract

**Introduction:**

Due to public concerns about deleterious biological consequences of radiofrequency electromagnetic fields (RF‐EMF), the potential effects of RF‐EMF on the central nervous system have received wide consideration.

**Methods:**

Here, two groups of C57BL/6 mice, aged 2 and 12 months, were exposed to 1,950‐MHz RF‐EMF at a specific absorption rate of 5.0 W/kg for chronic periods (2 hr/day and 5 days/week for 8 months). Behavioral changes were then assessed in the mice at 10 months (sham‐ or RF‐10M) and 20 months (sham‐ or RF‐20M), on the open‐field test, the Y‐maze test, and an object recognition memory task, while biological effects were analyzed via microarray gene profiling of the hippocampus.

**Results:**

Open‐field test results showed a decrease in the time duration spent at the center while there was a decrease in enhanced memory shown by the Y‐maze test and the novel object recognition test in the RF‐20M mice, compared to sham‐exposed mice, but no significant changes in the RF‐10M group. Based on a 2‐fold change cutoff, the microarray data revealed that 15 genes, which are listed as being involved in neurogenesis on Gene Ontology, were altered in both groups. Quantitative real‐time PCR for validation showed increased expression of *Epha8* and *Wnt6* in the hippocampi of RF‐20M group mice, although 13 additional genes showed no significant changes following RF‐EMF exposure.

**Conclusion:**

Therefore, cognitive enhancement following chronic exposure for 8 months to RF‐EMF from middle age may be associated with increases in neurogenesis‐related signals in the hippocampus of C57BL/6 mice.

## Graphical abstract

Young‐ and middle‐aged C57BL/6 mice, aged 2 and 12 months, were exposed to 1,950‐MHz RF‐EMF at an absorption rate of 5.0 W/kg for 2 hr/day and 5 days/week for 8 months. We found an association between chronic RF‐EMF exposure‐induced behavioral improvement and hippocampal gene signature in middle‐aged mice.

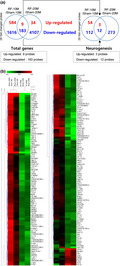

## 1 INTRODUCTION

As science and technology progress, exposure to radiofrequency electromagnetic fields (RF‐EMF) in daily life is increasing. In addition, the use of mobile phones and the ubiquity of Wi‐Fi have risen in recent years. Although RF‐EMF exposure during mobile phone use is within official safety standards, the public has raised concerns about the possible health risks associated with RF‐EMF.

In the year 2011, the International Agency for Research on Cancer (IARC) classified RF‐EMF as possibly carcinogenic to humans (Group 2B). In addition, the Scientific Committee on Emerging and Newly Identified Health Risks (SCENIHR) also reported on the potential effects of exposure to EMF. One study included in the SCENIHR report claimed that RF‐EMF exposure did not increase the risk of brain tumors, but some epidemiological studies raised questions about an increased risk of glioma and acoustic neuroma in heavy mobile phone users (Scientific Committee on Emerging Newly Identified Health Risks, 2015). Concerns continue to be expressed that long‐term exposure (>10 years) may increase the risk of intracranial tumors, primarily glioma (Prasad, Kathuria, Nair, Kumar, & Prasad, 2017).

In experimental animals, the potential risk of RF‐EMF exposure is still under investigation. It has been documented that RF‐EMF exposure impaired spatial cognitive function in rats, demonstrating the detrimental effects of RF‐EMF on the central nervous system in laboratory animals (Nittby, Grafström, et al., 2008). Other studies have reported that chronic RF‐EMF exposure did not affect spatial memory on radial‐arm maze or passive avoidance tests (Ammari et al., 2008; Dubreuil, Jay, & Edeline, 2003; Son et al., 2015). However, some beneficial effects were also reported. Previous studies have reported that chronic RF‐EMF exposure has positive effects on cognitive function in mice models of Alzheimer's disease (Banaceur, Banasr, Sakly, & Abdelmelek, 2013; Jeong et al., 2015; Son et al., 2018). Therefore, it remains unclear whether chronic RF‐EMF exposure influences brain function and associated behavioral changes. In addition, a previous report has reviewed the effects of RF exposure on cognitive behavior of experimental animals, suggesting that additional basic research is required with larger numbers of animals and standardized exposure system (Sienkiewicz & van Rongen, 2019). The precise mechanisms associated with brain function following chronic RF‐EMF exposure remain however unknown, and a more comprehensive study investigating behavioral changes in relation to hippocampal changes following chronic RF‐EMF exposure is needed. Therefore, this study was designed to examine neurobehavioral changes after chronic exposure to RF‐EMF in C57BL/6 mice.

Here, we assessed aspects of locomotor activity and hippocampus‐dependent memory in the open‐field test, the Y‐maze test, and an object recognition memory test after C57BL/6 mice at different ages were exposed to chronic RF‐EMF. Further, we aimed to identify potential responding genes in the whole cDNA of the mouse hippocampus after exposure to RF‐EMF.

## 2 MATERIALS AND METHODS

### 2.1 Animals

**FIGURE 1:**
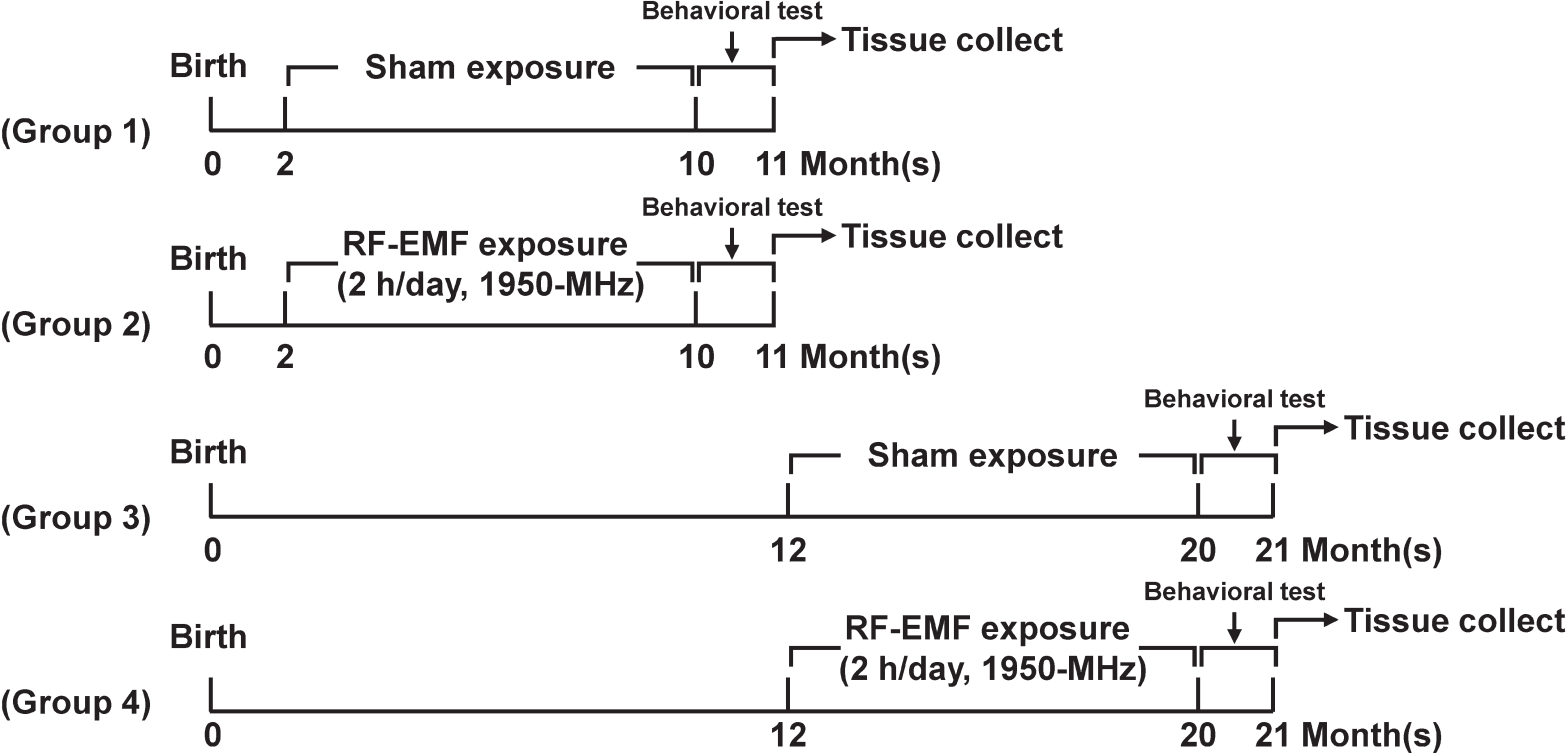
Schematic diagram of experimental procedure. The behavioral test and tissue collection timepoints from sham‐exposed and RF‐EMF‐exposed animals are shown. RF; RF‐EMF

Female C57BL/6J mice were obtained from Orient Biotech Co. Ltd. and were randomly assigned to four groups: (a) sham exposure, starting at 2 months (Sham‐10M [mice at 10 months of age after exposure] *n* = 12); (b) RF‐EMF exposure, starting at 2 months (RF‐10M, *n* = 12); (c) sham exposure, starting at 12 months (Sham‐20M [mice at 20 months of age after exposure], *n* = 12); and (d) RF‐EMF exposure, starting at 12 months (RF‐20M, *n* = 12) (Figure 1). In addition, 3‐month‐old mice (*n* = 2) were used as young animal controls for the microarray analysis. The animals were housed in a specific pathogen‐free facility controlled at 22 ± 2°C temperature and 60 ± 5% humidity with a 12:12‐hr light/dark cycle with access to a normal diet and autoclaved water ad libitum. All animal experimental protocols were approved by the Korea Institute of Radiological and Medical Sciences (KIRAMS) Institutional Animal Care and Use Committee (IACUC permit number: KIRAMS2014‐0055/2017‐0053) and performed in accordance with the guidelines specified by that committee.

### 2.2 Whole‐body RF exposure system

The RF‐EMF exposure system, designed for in vivo experiments, was installed in a reverberation chamber (ERE‐MRC‐1.5; ERETEC). System description, the uniformity of the field dose, and the specific absorption rate (SAR) were in accordance with previous reports (Lee et al., 2012; Son et al., 2018). Briefly, the 1,950‐MHz RF‐EMF signal was generated using a microprocessor unit (MPU) chip on which WCDMA‐formatted code controlled a central processing unit (CPU). Subsequently, the signal was amplified using an additional high‐power amplifier (PCS60WHPA_CW; Kortcom) after it was passed through a separate digital attenuator. An 11‐bit digital PIN diode attenuator (Model 349; General Microwave) was used to control the output power level (maximum: 60 W). The transmitting antennae were commercially purchased (patch type, KCAN1900PA; Korea Telecommunication Components), and a computer was used to control the exposure level and time. The external dimensions of the reverberation chamber were 2,295 × 2,293 × 1,470 mm; the walls were made of stainless steel with a thickness of 2.3 mm. To measure field uniformity, five cages were placed in the test area, and the field strength was measured for 1 min at each of 24 points. The distribution of the electric field inside the chamber was determined using a three‐axis isotropic probe (HI‐6005; ETS‐Lindgren). Differences in field distribution were much <3 dB. The SAR distribution for each caged mouse was calculated using a mouse phantom (Chungnam National University, Daejon, Korea); the simulation featured 40 tissues and a voxel size of 1 mm. The power output was controlled at 52 W to achieve an average whole‐body SAR of 5 W/kg. The reverberation chamber was placed in the animal facility, and the ventilation, temperature, and humidity were controlled. The animals were exposed to the 1,950‐MHz RF‐EMF according to the following schedule: Whole‐body average SAR 5 W/kg, 2 hr/day, 5 days/week, for 8 months. RF‐EMF exposure time was alternated every other week in the morning (09:00–11:00) and in the afternoon (14:00–16:00). The mice of the sham‐exposure group were placed inside the chambers during the same period without RF‐EMF signals being applied. During the RF exposure, the air temperature inside the test area was maintained at 20 ± 3°C and the mice were able to move freely. Body temperature was measured as rectal temperature before and immediately after RF‐EMF exposure, and did never increase by more than 0.5°C.

### 2.3 Behavioral testing

Behavioral testing was conducted with after chronic (8 months) RF‐EMF exposure starting at 2 and 12 months of age, respectively (Figure 1). The order of behavioral testing is open‐field, object recognition memory test, followed by Y‐maze test.

#### 2.3.1 Open‐field test

Mice were habituated to the testing room for 30 min before any behavioral experiment. All behavioral testing was conducted during daytime (between 14:00 and 18:00). Behavioral experiments were conducted by a researcher blind to the groups and between each session, the boxes were cleaned with ethanol spray to remove odor effect. The open‐field test was used to assess general activity. The mice were placed in the central area of the acrylic chamber (45 × 45 × 30 cm) and allowed to freely move in the open‐field apparatus without restrictions for 10 min. The footpath of all animals was recorded with an automated video‐tracking system using Viewer3 program (http://www.biobserve.com, Biobserve Viewer, RRID:SCR_014337). The tracking system was then used to quantify the animals' total distance traveled, percentage of activity, and time spent in center zone.

#### 2.3.2 Y‐maze test

Descriptions of the Y‐maze test have been provided previously (Son et al., 2016). The Y‐maze test was used to measure working memory and reference memory, which were assessed by recording spontaneous alternation behavior. Activity was recorded for 8 min and analyzed with a computer program (Viewer3). Alternation was defined as successive entries into the three different arms on overlapping triplet sets and calculated as the ratio of actual to possible alternations (defined as the total number of arm entries minus two) multiplied by 100, as follows: % alternation = (number of alternations)/(total arm entries − 2) × 100.

#### 2.3.3 Novel object recognition memory test

The novel object recognition memory test was conducted according to a previous study (Son et al., 2016). Briefly, mice were individually placed into a square acrylic chamber (45 × 45 × 30 cm) for habituation. All apparatus and objects were sprayed with 70% (v/v) ethanol between each experiment to remove odor cues. On training day, two of the same objects were presented to each mouse for 10 min. At 24 hr after training, one familiar object and one novel object were placed into the chamber for testing. Testing session was conducted for 10 min. The camera was placed above the chamber for video tracking (Viewer3). Preference for the novel object was expressed as a percentage of total exploration time for the object (the exploration time for the novel object divided by the time the animal spent interacting with both objects).

### 2.4 Sample preparation

Following the behavioral test, mice were euthanized by intraperitoneal injection of 30 mg/kg tiletamine–zolazepam (Zoletil; Virbac) and 10 mg/kg xylazine (Rompun; Bayer Korea). The mouse brain tissue was taken out from the brain skull, and the hippocampus region was dissected with microforceps. The hippocampi were kept individually in a sterile tube and stored immediately at −80°C until analysis was performed.

### 2.5 cDNA microarray

Microarray analysis (*n* = 1 for sham‐10M and RF‐10M group, *n* = 2 for sham‐20M group, and *n* = 3 for RF‐20M group) was conducted as per a previous study (Jeong et al., 2015). For the negative control for normal hippocampus, we used the hippocampi from 3‐month‐old mice (*n* = 2). To synthesize target cRNA probes and hybridization, microarrays were performed using Agilent Mouse 44K whole‐genome expression array with Low Input QuickAmp Labeling kit (Agilent Technology), following the manufacturer's protocols, at eBiogen. Averages of normalized ratios were calculated by dividing the average test normalized signal intensity by the average control normalized signal intensity.

### 2.6 Bioinformatic analysis

Up‐ or downregulated genes were identified using ExDEGA v1.2.1.0 (http://www.e‐biogen.com, eBiogen Inc.): a fold change above 2 was defined as “upregulated” while one lower than 0.5 means “downregulated.” A hierarchical cluster analysis was performed using Euclidean distance metric and average linkage. The hierarchical clustering and gene expression heat map were visualized using the Multi Experiment Viewer software program v4.9 (http://mev.tm4.org, MeV). The functional classification of the genes and gene ontology (GO) classification was based on a search performed using DAVID Bioinformatics Resources (http://david.abcc.ncifcrf.gov, DAVID, RRID:SCR_001881).

### 2.7 Quantitative Real‐Time PCR (qRT‐PCR)

Total RNA was isolated using Qiazol Lysis Reagent (#79306, Qiagen) and reverse transcribed into cDNA using an amfiRivert cDNA Synthesis Platinum Master Kit (R5600, GenDEPOT), as per manufacturer's instructions. For qRT‐PCR analysis, hippocampal tissues were used for each group (*n* = 5/group). The qRT‐PCR was performed using gene‐specific primers and the qPCR SYBR Green 2X Mastermix kit (#18303, MBiotech). Glyceraldehyde 3‐phosphate dehydrogenase (GAPDH) was used as a housekeeping gene. The sequences for forward and reverse primers were the following: *GAPDH* F: 5′‐CAAGAAGGTGGTGAAGCAGG‐3′ and R: 5′‐AGGTGGAAGAGTGGGAGTTG‐3′; *Wnt6* F: 5′‐GGACATCCGAGAGACAGCTT‐3′ and R: 5′‐TGCCTGACAACCACACTGTA‐3′; *Epha8* F: 5′‐GTTGTGACCTCAGCTACTACC‐3′ and R: 5′‐ACAGACGTTCCATTTACGCT‐3′. The sequences for 13 additional genes are shown in Table S1. The qRT‐PCR experiments were performed on a CFX96TM Real‐Time PCR System (Bio‐Rad).

### 2.8 Statistical analyses

All data are presented as the mean ± standard error of the mean (*SEM*). The data except from the open‐field test were analyzed by two‐tailed Student's *t* tests implemented in GraphPad Prism 7.0 (GraphPad software). For open‐field test, nonrepeated‐measures two‐way ANOVA was used to compare between groups in the open‐field test. Holm–Sidak post hoc tests were applied for multiple comparisons where warranted by a significant omnibus *F*‐statistic. In all analyses, *p* < .05 was used to indicate statistical significance.

## 3 RESULTS

### 3.1 Effect of chronic RF exposure on locomotor activity

**FIGURE 2:**
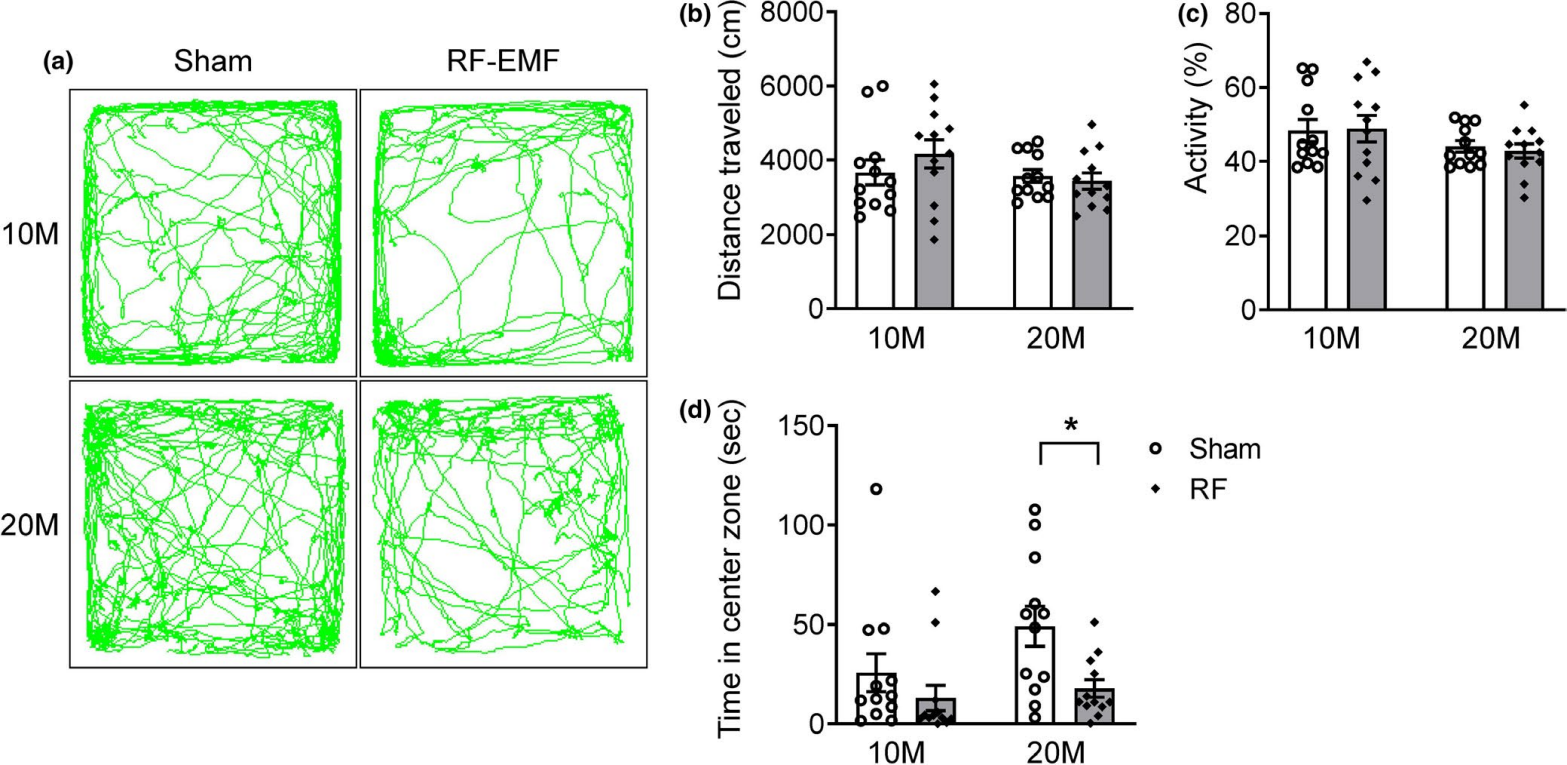
Effect of chronic RF‐EMF exposure on basal locomotor activity in mice of different ages. General activity was measured with the open‐field test in 10M (months) and 20M mice following sham or RF‐EMF exposure, respectively. (a) Representative traces of mouse movements during the open‐field test. The total distance traveled (b), activity (c), and time spent in the center zone (d) were measured in the 10M and the 20M group. Values represent the mean ± *SEM* (*n* = 12). Statistical significance has been defined as *p < .05 Sham versus RF group. RF; RF‐EMF

To investigate the effect of long‐term RF‐EMF exposure on behavioral changes, two groups of C57BL/6 mice, young adult‐aged (2 months) and middle‐aged (12 months), respectively, were exposed, at different time points, to 1,950‐MHz RF‐EMF at a whole‐body average SAR of 5.0 W/kg for 2 hr/day and 5 days/week for 8 months (Figure 1). To confirm whether chronic RF‐EMF exposure affected locomotor activity in the mice of different ages, we first measured basic locomotor activity in a novel environment via the open‐field test. The open‐field behavior test indicated no significant changes in locomotor activity (measured as the total distance traveled, activity, and the time spent in the center zone) in the RF‐10M group when compared with sham‐10M controls (Figure 2a–d). The time spent in the center tended to be longer in the sham‐20M compared to the sham‐10M group, although the difference between the groups was not statistically significant. However, a significant decrease in time spent in the center zone was evident in the RF‐20M group compared to sham‐20M controls (*p* = .036, Sham‐20M vs. RF‐20M; Figure 2d).

### 3.2 Impact of chronic RF exposure on memory function

**FIGURE 3:**
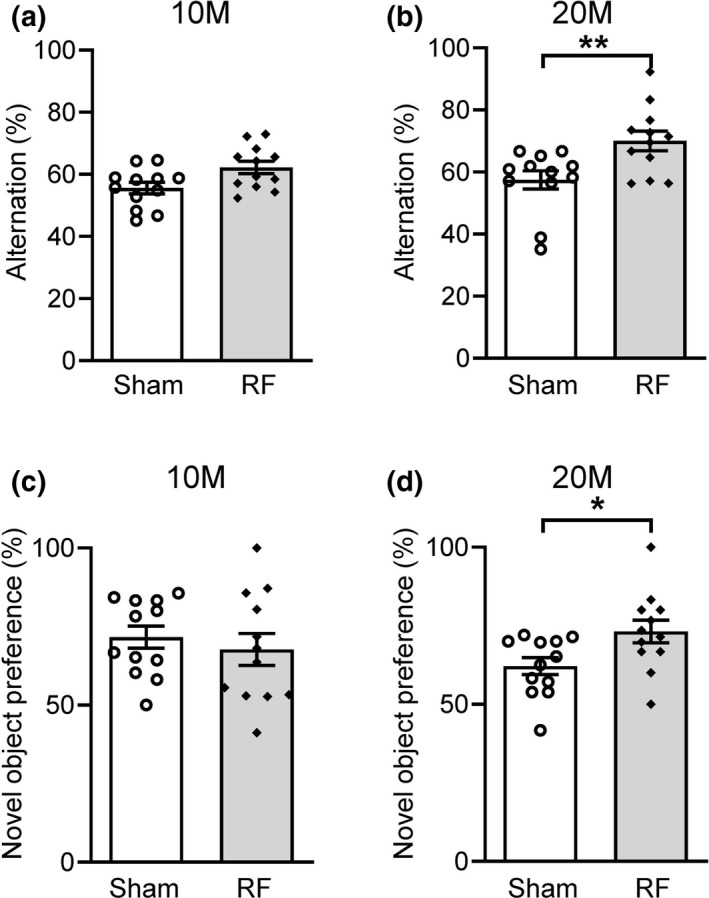
Y‐maze and object recognition memory test after chronic RF‐EMF exposure in mice of different ages. The Y‐maze test was used to evaluate spatial memory in 10M (months) (a) and 20M (b) mice. Alternation percentages indicate the frequency of nonoverlapping entries compared to the total number of entries into the three arms. (c and d) RF‐10M and RF‐20M groups exhibited equal preferences for the two test objects during training compared with age‐matched controls (data not shown). Novel object recognition was significantly improved in the 20M mice after chronic RF‐EMF exposure (d). Values represent the mean ± *SEM* (*n* = 12). Statistical significance has been defined as *p < .05 and **p < .01 Sham versus RF group. RF; RF‐EMF

We examined whether chronic RF‐EMF exposure affects spatial memory in C57BL/6 mice using the Y‐maze test. The RF‐10M mice that had been exposed to RF‐EMF for 8 months showed no differences in spontaneous alternations on the Y‐maze test (Figure 3a), while a significant increase in spontaneous alternation was observed in RF‐20M mice compared to the age‐matched sham‐exposed group (*p* = .008, Sham‐20M vs. RF‐20M; Figure 3b).

To evaluate hippocampus‐related nonspatial memory function, an object recognition memory test was performed in the mice exposed to 8 months of RF‐EMF. Twenty‐four hours after the training session, the animals were tested for novel object recognition. RF‐10M mice showed no significant differences in their preference for the novel object (Figure 3c). However, chronic exposure to RF‐EMF significantly increased the mice's preference for the novel object in the RF‐20M group (*p* = .023, Sham‐20M vs. RF‐20M; Figure 3d). These results indicate that learning and memory functions, as examined on the Y‐maze and a novel object recognition memory test, were improved after 8 months of RF‐EMF exposure from a middle age.

### 3.3 Differentially expressed genes identified by microarray analysis in the hippocampi of RF‐EMF‐exposed mice

**FIGURE 4:**
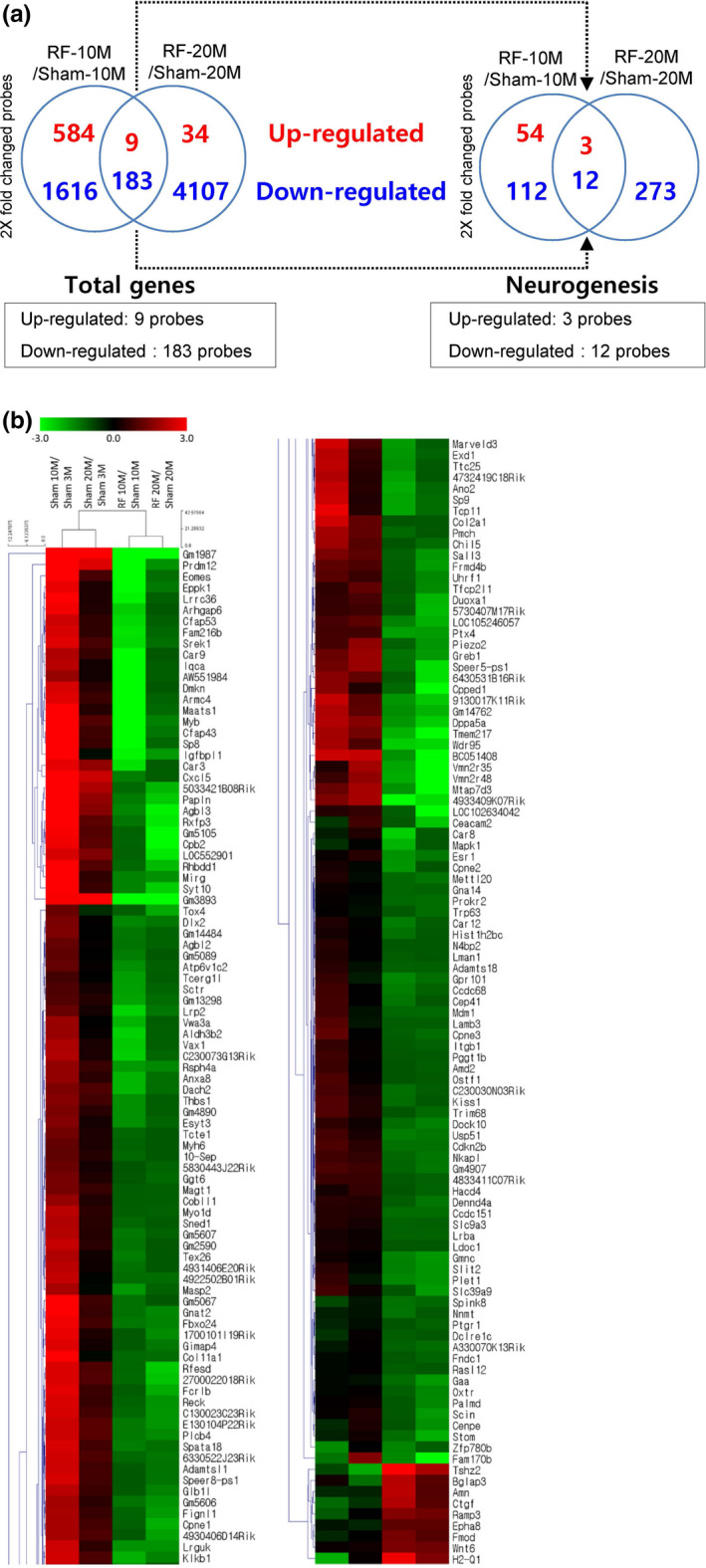
Differentially expressed genes after chronic RF‐EMF exposure. (a) Venn diagrams for 8‐month RF‐EMF exposure showing genes that were up‐ or downregulated in both 10M (months) and 20M groups. (b) Heat map of genes differentially expressed between the sham‐ and RF‐EMF‐exposed mice groups. RF; RF‐EMF

**FIGURE 5:**
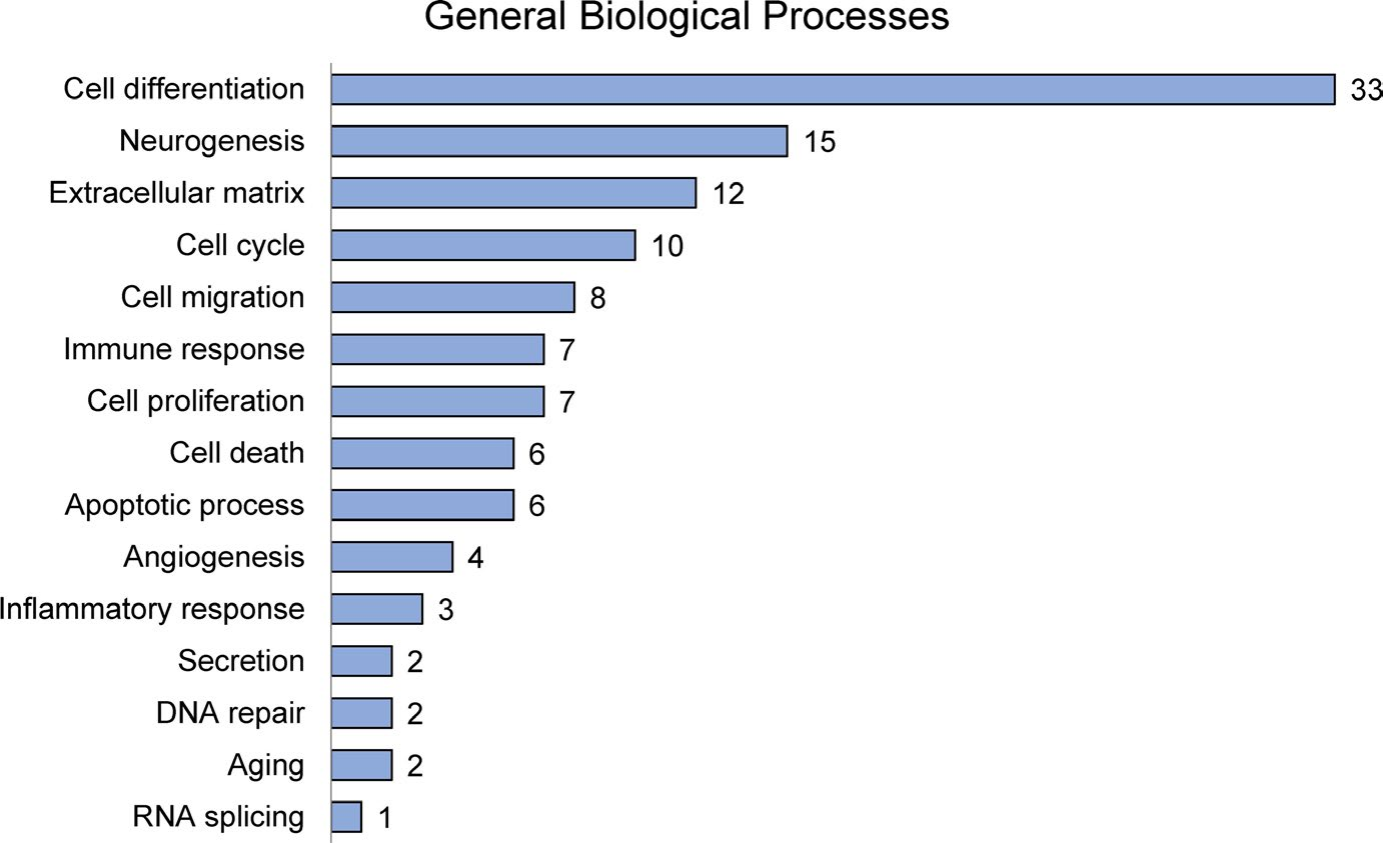
Gene Ontology analysis in the mouse hippocampus after chronic RF‐EMF. The number of genes that could be classified into the fifteen different categories in general biological processes is indicated

To elucidate differences in gene expression in RF‐exposed and sham‐exposed mice, the Agilent Mouse 44K whole‐genome expression array was utilized using hippocampal tissue. The expression of transcripts from the hippocampi of the two sham‐exposed groups was compared to the expression of the two RF‐exposed groups of different ages. Hippocampal genes that were differentially regulated and expressed after chronic RF‐EMF were analyzed using the DAVID‐GO bioinformatics subroutine with fold changes. Based on a 2‐fold change cutoff, the microarray identified 192 genes modulated by RF exposure in both the RF‐10M and RF‐20M group (Figure 4a, Table S2). Most hippocampal genes identified were downregulated following chronic RF exposure (Figure 4b). The 192 RF‐specific genes were related to diverse biological processes such as “cell differentiation” (33 genes), “neurogenesis” (15 genes), “extracellular matrix” (12 genes), and “cell cycle” (10 genes) (Figure 5). More specifically, among the classified genes related to neurogenesis, three genes were upregulated while 12 genes were downregulated (Table 1). To explore potential interactions between selected genes, we have constructed an extended PPI network of genes through STRING database and visualized by Cytoscape (Figure S1).

**TABLE 1:** Expression fold changes from microarray data

Gene Symbol	Gene Name	Accession No.	Sham 10M/Sham 3M	Sham 20M/Sham 3M	RF 10M/Sham 10M	RF 20M/Sham 20M
Wnt6	wingless‐type MMTV integration site family, member 6	NM_009526	1.087	1.145	2.782	2.474
Epha8	Eph receptor A8	NM_007939	0.843	0.742	2.144	2.135
Fmod	fibromodulin	NM_021355	0.736	1.024	2.669	2.016
Mapk1	mitogen‐activated protein kinase 1	NM_001038663	0.676	0.970	0.231	0.490
Cxcl5	chemokine (C‐X‐C motif) ligand 5	NM_009141	8.350	5.310	0.353	0.470
Vax1	ventral anterior homeobox 1	NM_009501	4.056	1.255	0.195	0.459
Itgb1	integrin beta 1 (fibronectin receptor beta)	NM_010578	1.537	1.073	0.476	0.453
Dlx2	distal‐less homeobox 2	NM_010054	2.691	1.028	0.335	0.378
Gnat2	guanine nucleotide binding protein, alpha transducing 2	NM_008141	8.322	1.589	0.392	0.331
Slit2	slit homolog 2 (Drosophila)	NM_001291227	1.428	0.959	0.351	0.304
Sall3	sal‐like 3 (Drosophila)	NM_178280	2.792	2.134	0.448	0.294
Cpne1	copine I	NM_170588	3.946	1.767	0.448	0.237
Duoxa1	dual oxidase maturation factor 1	NM_001305262	1.536	1.821	0.457	0.225
Prdm12	PR domain containing 12	NM_001123362	131.708	6.083	0.116	0.303
Eomes	eomesodermin homolog (*Xenopus laevis*)	NM_010136	19.424	1.827	0.062	0.406

### 3.4 Changes in the levels of differentially expressed, neurogenesis‐specific genes

**FIGURE 6:**
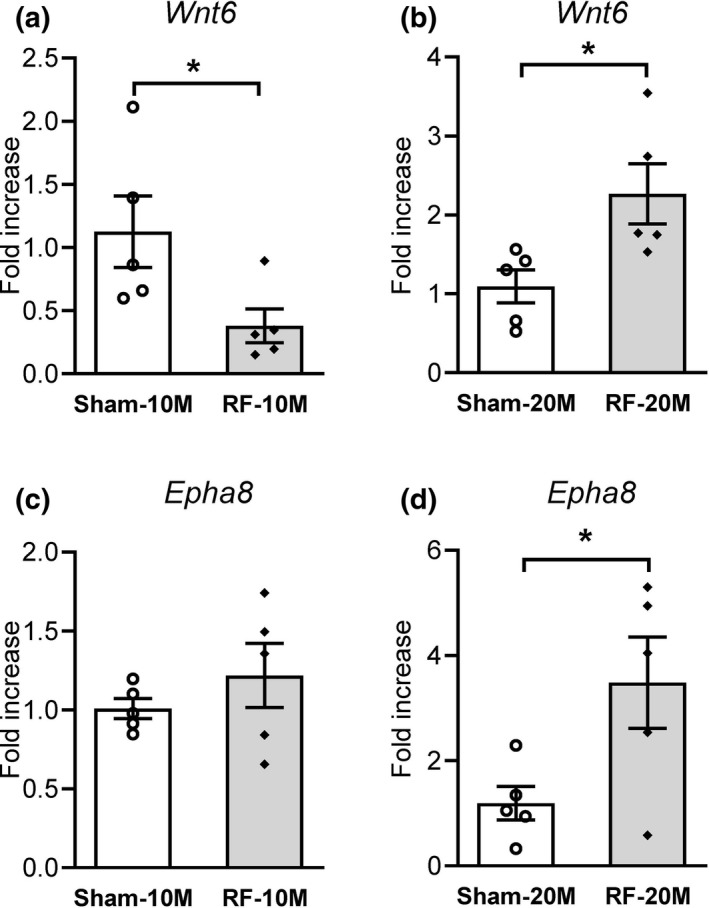
Changes in Wnt6 and Epha8 levels in the mouse hippocampus after chronic RF‐EMF. The bar graphs show the levels of mRNA encoding *Wnt6* in the hippocampi of 10M (months) (a) and 20M (b) mice, respectively. The mRNA levels of *Epha8* were evaluated in the mice hippocampi of 10M (c) and 20M (d) mice. All data are reported as mean ± *SEM* (*n* = 5 per group). Statistical significance has been defined as *p < .05 Sham versus RF group

The qRT‐PCR analysis confirmed the expression profile for 15 neurogenesis‐specific genes (Figure 6 and Figure S2). Whereas *Wnt6* levels in RF‐10M mice were decreased significantly (*p* = .045, Sham‐10M vs. RF‐10M; Figure 6a), a significant increase in *Wnt6* expression was observed in the RF‐20M group compared to age‐matched sham‐exposed mice (*p* = .028, Sham‐20M vs. RF‐20M; Figure 6b). Similar to *Wnt6*, the expression level of *Epha8* increased significantly in RF‐20M mice (*p* = .038, Sham‐20M vs. RF‐20M; Figure 6d). However, the *Epha8* levels in RF‐10M mice did not differ from those in sham‐exposed controls (Figure 6c). In addition, 13 genes except *Wnt6* and *Epha8* showed no significant changes following RF‐EMF exposure (Figure S2). These findings demonstrate a marked increase in the mRNA expression of *Wnt6* and *Epha8* following chronic RF‐EMF in middle‐aged mice.

## 4 DISCUSSION

This study aimed to demonstrate the effects of chronic RF‐EMF exposure on behavioral function and hippocampal gene expression. We found that chronic RF‐EMF exposure did not induce behavioral changes in young adult mice but improved cognitive function in middle‐aged mice. Furthermore, altered neurogenesis‐related gene expression was found in C57BL/6 mice after chronic RF‐EMF exposure from a middle age.

The rapid development and expansion of mobile communication in everyday life has led to concerns about health problems induced by exposure of the body, and especially the central nervous system, to RF‐EMF. A previous report found that subchronic RF‐EMF exposure (SAR: 6 W/kg, 5 days/week for 2 months) may adversely affect brain function by increasing glial fibrillary acidic protein (GFAP) expression (Ammari et al., 2010). Other reports have however found beneficial or no effects of RF‐EMF exposure on cognitive function (Barth et al., 2008; Son et al., 2016). In previous studies, the beneficial effects of RF‐EMF exposure were usually observed in mice models of Alzheimer's disease, indicating that RF‐EMF might affect AD pathology (Arendash et al., 2010; Banaceur et al., 2013; Jeong et al., 2015). Interestingly, researchers have focused on the possible effects of RF‐EMF on the senescent brain. A previous report found no differences in learning capabilities and behavior after RF‐EMF exposure (SAR: 6 W/kg, 45 min/day for 2 months) in young (4–6 months) or old (22–24 months) rats (Bouji, Lecomte, Gamez, Blazy, & Villegier, 2016). Another study reported that adult (5 months) mice showed cognitive benefits after 5 months of RF‐EMF exposure, indicated by cognitive interference test results (Arendash et al., 2010). It thus remains controversial whether RF‐EMF exposure has positive or negative effects on the brain functions of senescent animals. A more comprehensive study investigating the potential effect of RF‐EMF exposure on the aging central nervous system is therefore needed. In this study, we applied whole‐body average SAR 5 W/kg which was much higher than the everyday exposure limit of whole‐body average SAR 0.08 W/kg set by International Commission on Non‐Ionizing Radiation Protection (ICNIRP). Despite this high exposure level, it did not alter mice body temperature, which allowed us to investigate the beneficial effects of RF‐EMF exposure with high SAR on the brain of adult and aged mice. A previous report has demonstrated increases in the time spent in the center in the open‐field test in aged compared to young mice (Shoji & Miyakawa, 2019). Consistent with this previous study, our sham‐20M mice tended to spend longer time in the center area in the open‐field test. While we found no behavioral changes in locomotor activity and cognitive function in mice exposed to RF‐EMF from a young age, RF‐EMF exposure resulted in protection against age‐related changes in center time in the RF‐20M group compared to age‐matched sham‐exposed controls. In addition, our 20M mice exhibited improved performance on the object recognition memory test and the Y‐maze test after chronic RF‐EMF exposure, compared to sham‐exposure controls, indicating that such beneficial effects of chronic RF exposure occur when middle‐aged mice, not young‐aged mice, are chronically exposed to RF‐EMF. However, since we used extremely high SAR, further dose–response studies are required to verify the biological effects of RF‐EMF on brain functions.

To demonstrate potential interactions between RF exposure and neurobiological systems, several large‐scale analysis studies have focused on the impact of RF‐EMF on gene expression, in an effort to identify responding genes (Jeong et al., 2015; Leszczynski et al., 2012; McNamee et al., 2016; Yang et al., 2012). It has been reported earlier that no significant differences were found in gene expression in the hippocampus or cortex for any of 31,099 gene targets after 1.8‐GHz GSM‐modulated RF exposure (SAR: 13 mW/kg) for 6 hr (Nittby, Widegren, et al., 2008). Another study reported that 75 genes were differentially expressed in the whole brain of Balb/c mice following RF exposure (SAR: 1.1 W/kg) for 1 hr, although these differences were not confirmed by real‐time PCR (Paparini et al., 2008). While previous studies have examined the effects of RF exposure on a variety of cellular functions, no study has been conducted to analyze gene expression changes induced by chronic RF exposure. We thus performed a microarray analysis using the mice exposed to RF from young age or from middle age to identify gene profiles in hippocampal tissue. Of 39,429 genes, nine upregulated and 183 downregulated (more than 2‐fold up or down) genes were commonly observed in both groups. Among these 192 genes, fifteen GO categories were analyzed in both the RF‐10M and the RF‐20M group after the exposure (Figure 5). Notably, ~8% (15 genes) were characterized as neurogenesis‐related, that is, involved in neuronal and behavioral functions. However, comprehensive further microarray studies are needed to evaluate the mechanisms underlying electromagnetic field action on biology.

The hippocampus is located within the ventromedial aspect of the temporal cortex in the brain and plays important roles in memory and emotional regulation. It has previously been suggested that neurogenesis in the hippocampus might play a pivotal role in hippocampus‐dependent functions, including learning and memory and emotion regulation (Sahay & Hen, 2007; Shors et al., 2001). The current study extends the analysis of potential responding genes in mice following in vivo RF field exposure by analyzing neurogenesis‐related gene expression in the mouse hippocampus. Among the 192 genes (nine upregulated and 183 downregulated) that were differentially expressed after RF exposure, 15 genes (three upregulated and 12 downregulated), which were related to neurogenesis in gene ontology, were selected for further verification of the microarray analysis using qRT‐PCR. Of these differential expression genes, *Wnt6* and *Epha8* were notable since their expression levels were significantly increased in the RF‐20M group compared to age‐matched sham controls. Previous studies have shown that Wnt signaling, including *Wnt6*, and Eph/ephrin signaling, including *Epha8*, influence the regulation of synapse formation, function and plasticity (Fortress & Frick, 2016; Gu et al., 2005; Yang, Wei, Chen, & Wu, 2018). Previous reports have shown that *Epha8* is implicated in neurite growth (Buchser, Slepak, Gutierrez‐Arenas, Bixby, & Lemmon, 2010). In addition, overexpression of *Wnt6* ameliorates synaptic and behavioral deficits in mice model of Rett syndrome (Hsu, Ma, Liu, Tai, & Lee, 2020). Thus, the increased expression of *Wnt6* and *Epha8* may be correlated with the occurrence of cognitive enhancements following chronic RF exposure from a middle age. However, the decreased expression of *Wnt6*, found in RF‐10M group, was not correlated with the data in microarray. In the present study, the microarray and qRT‐PCR results do not correlate well; this might be due to data normalization processes, different sequences of the primers used, and methodological biases that might have influenced the results. Further studies are warranted to verify the specific mechanisms underlying neurogenesis‐related gene expression by PCR and immunostaining and the associated behavioral functions following chronic RF‐EMF exposure.

## 5 CONCLUSION

Our study, although intended as a preliminary investigation to identify the effect of chronic RF‐EMF on behavior in young or middle‐aged mice, yields very interesting results. Behavioral changes were found only when middle‐aged mice were exposed to chronic RF‐EMF. We identified *Wnt6* and *Epha8* as the genes specifically altered following RF‐EMF exposure, but the increase in these two genes was confirmed only in middle‐aged mice (20M). Our results thus demonstrate an association between chronic RF‐EMF exposure‐induced behavioral improvement and increased hippocampal gene expression of *Wnt6* and *Epha8* in middle‐aged mice. These findings support an effect of RF‐EMF on brain function and suggest new research targets, but future studies need to elucidate the underlying biological mechanisms.

## CONFLICT OF INTEREST

The authors declare no competing interests.

## AUTHOR CONTRIBUTION

Y.J.J. and Y.S. performed the behavioral studies and molecular analyses, analyzed the data, and wrote the manuscript. H.D.C., N.K., and Y.S.L. designed the experiments and analyzed the data. Y.G.K. and H.J.L. conceived and designed the experiments, analyzed the data, and wrote the manuscript.

### Peer Review

The peer review history for this article is available at https://publons.com/publon/10.1002/brb3.1815.

## Supporting information

Appendix S1

## Data Availability

All data generated or analyzed during this study are included in this published article (and its Supplementary files).
