# Supplementary material for: Behavioral changes and gene profile alterations after chronic 1,950‐MHz radiofrequency exposure: An observation in C57BL/6 mice
Source: Brain Behav. 2020 Aug 28;10(11):e01815. doi: 10.1002/brb3.1815 (PMC7667305; doi:10.1002/brb3.1815)
Supplement: Supplementary file 1 — Appendix S1 [file BRB3-10-e01815-s001.docx]

Appendix S1

Behavioral changes and gene profile alterations after chronic 1950-MHz radiofrequency exposure: An observation in C57BL/6 mice

Ye Ji Jeong^1,2,+^, Yeonghoon Son^1,3,+^, Hyung-Do Choi^4^, Nam Kim^5^, Yun-Sil Lee^6^, Young‐Gyu Ko^2,*^, and Hae-June Lee^1,*^

^1^Division of Basic Radiation Bioscience, Korea Institute of Radiological & Medical Sciences, Seoul, Korea

^2^Division of Life Sciences, Korea University, Seoul, Korea

^3^Primate Resources Center, Korea Research Institute of Bioscience and Biotechnology (KRIBB), Jeonbuk, Korea

^4^Department of EMF Research Team, Radio and Broadcasting Technology Laboratory, ETRI, Daejon, Korea

^5^School of Electrical and Computer Engineering, Chungbuk National University, Cheongju, Chungbuk, Korea

^6^Graduate School of Pharmaceutical Sciences, Ewha Womans University, Seoul, Korea

+These authors contributed equally to this work.

*Corresponding authors:

Young‐Gyu Ko, PhD, Division of Life Sciences, Korea University, Seoul 02841, Korea; Tel: +82-2-3290-3453; Fax: +82-2-927-9028; E‐mail: ygko@korea.ac.kr

Hae-June Lee, DVM, PhD, Division of Basic Radiation Bioscience, Korea Institute of Radiological & Medical Sciences, Seoul 01812, Korea; Tel: +82-2-970-1638; Fax: +82-2-970-1985; E-mail: hjlee@kirams.re.kr

**Table S1.** Primer sequences for real-time qPCR analysis

| Gene Name | Primer sequence | |
| --- | --- | --- |
| Cpne1 | Forward | 5’-CCCGGCTGAGTTTGAATGTG-3’ |
|  | Reverse | 5’-GCAGCCTCCCATCACATAGT-3’ |
| Cxcl5 | Forward | 5’-GGTTCCATCTCGCCATTCAT-3’ |
|  | Reverse | 5’-TTAAGCAAACACAACGCAGC-3’ |
| Dlx2 | Forward | 5’-GTCTCCTACTCCGCCAAAAG-3’ |
|  | Reverse | 5’-TTCGGATTTCAGGCTCAAGG-3’ |
| Duoxa1 | Forward | 5’-TCACTCATATCGCCCTGTCC-3’ |
|  | Reverse | 5’-TGACCAGGCCAAGCAGAATA-3’ |
| Eomes | Forward | 5’-TCGTGGAAGTGACAGAGGAC-3’ |
|  | Reverse | 5’-AGCTGGGTGATATCCGTGTT-3’ |
| Fmod | Forward | 5’-GATCCCTCCTGTCAACACCA-3’ |
|  | Reverse | 5’-CGCTTGATCTCGTTCCCATC-3’ |
| Gnat2 | Forward | 5’-CTACCTCCCTAACGAGCAGG-3’ |
|  | Reverse | 5’-TCCTCTCTGATCTCTGCCCT-3’ |
| Itgb1 | Forward | 5’-ACTGGTCCATGTCTAGCGTC-3’ |
|  | Reverse | 5’-CATGTCTCACAAGTTGGCCC-3’ |
| Mapk1 | Forward | 5’-CCCGTGTTGCAGATCCAGAT-3’ |
|  | Reverse | 5’-TGCAGCCCACAGACCAAATA-3’ |
| Pdrm12 | Forward | 5’-CATCAAGTGTGCCCGGAATG-3’ |
|  | Reverse | 5’-TCCTGGTCTGGAGGGATCAT-3’ |
| Sall3 | Forward | 5’-CATCCAGCACTGTAGGCAAC-3’ |
|  | Reverse | 5’-TGAGGTAGCAGAGGGTTTGG-3’ |
| Slit2 | Forward | 5’-CGGATTTGCTAGAGGGTTCG-3’ |
|  | Reverse | 5’-CGACTTCAACTTGGCGGTAA-3’ |
| Vax1 | Forward | 5’-GAGTTCCAGCGTTGCCAATA-3’ |
|  | Reverse | 5’-CTTCTTCTGCTTAGTCCGCC-3’ |
| GAPDH | Forward | 5’-CAAGAAGGTGGTGAAGCAGG-3’ |
|  | Reverse | 5’-AGGTGGAAGAGTGGGAGTTG-3’ |

**Table S2.** The list of 2 folds up- or down-regulated genes in the RF exposed mouse hippocampus

| GeneSymbol | GeneName | Genbank Accession | Sham 10M/ Sham3M | Sham 20M/ Sham3M | RF 10M/ Sham10M | RF 20M/ Sham20M |
| --- | --- | --- | --- | --- | --- | --- |
| Prdm12 | PR domain containing 12 | NM_001123362 | 131.708 | 6.083 | 0.116 | 0.303 |
| Gm1987 | predicted gene 1987 | NM_001193667 | 117.184 | 176.840 | 0.064 | 0.011 |
| Papln | papilin, proteoglycan-like sulfated glycoprotein | NM_001205343 | 17.845 | 3.341 | 0.366 | 0.195 |
| 5033421B08Rik | RIKEN cDNA 5033421B08 gene | XR_872469 | 12.588 | 4.169 | 0.423 | 0.226 |
| Agbl3 | ATP/GTP binding protein-like 3 | NM_001289656 | 11.234 | 3.060 | 0.331 | 0.139 |
| Cxcl5 | chemokine (C-X-C motif) ligand 5 | NM_009141 | 8.350 | 5.310 | 0.353 | 0.470 |
| Rxfp3 | relaxin family peptide receptor 3 | NM_178717 | 8.340 | 2.142 | 0.379 | 0.149 |
| Gm5105 | predicted gene 5105 | NR_037975 | 7.799 | 2.006 | 0.448 | 0.130 |
| Gm3893 | predicted gene 3893 | NR_033506 | 7.684 | 9.559 | 0.118 | 0.062 |
| Cpb2 | carboxypeptidase B2 (plasma) | NM_019775 | 7.244 | 2.114 | 0.441 | 0.128 |
| Car3 | carbonic anhydrase 3 | NM_007606 | 6.670 | 3.313 | 0.158 | 0.469 |
| LOC552901 | uncharacterized LOC552901 | AK035826 | 5.778 | 2.910 | 0.452 | 0.149 |
| Col2a1 | collagen, type II, alpha 1 | NM_001113515 | 5.580 | 2.222 | 0.492 | 0.485 |
| Plcb4 | phospholipase C, beta 4 | AK036896 | 5.349 | 2.076 | 0.367 | 0.323 |
| BC051408 | cDNA sequence BC051408 | XR_883967 | 5.345 | 5.099 | 0.303 | 0.097 |
| E130104P22Rik | RIKEN cDNA E130104P22 gene | AK053520 | 5.201 | 2.085 | 0.370 | 0.296 |
| 9130017K11Rik | RIKEN cDNA 9130017K11 gene | AK013327 | 5.064 | 2.068 | 0.292 | 0.193 |
| Dppa5a | developmental pluripotency associated 5A | NM_025274 | 4.319 | 2.989 | 0.294 | 0.185 |
| Gm14762 | predicted gene 14762 | AK052035 | 4.185 | 2.175 | 0.314 | 0.232 |
| Pmch | pro-melanin-concentrating hormone | NM_029971 | 3.836 | 2.261 | 0.420 | 0.490 |
| Tmem217 | transmembrane protein 217 | NM_001162901 | 3.800 | 2.595 | 0.227 | 0.128 |
| Chil5 | chitinase-like 5 | XM_006502524 | 3.712 | 2.042 | 0.492 | 0.414 |
| 6430531B16Rik | RIKEN cDNA 6430531B16 gene | AK164921 | 3.062 | 2.266 | 0.461 | 0.163 |
| 4933409K07Rik | RIKEN cDNA 4933409K07 gene | NR_033123 | 2.800 | 3.962 | 0.121 | 0.166 |
| Sall3 | sal-like 3 (Drosophila) | NM_178280 | 2.792 | 2.134 | 0.448 | 0.294 |
| Speer5-ps1 | spermatogenesis associated glutamate (E)-rich protein 5, pseudogene 1 | NR_001582 | 2.532 | 3.492 | 0.397 | 0.160 |
| Greb1 | gene regulated by estrogen in breast cancer protein | NM_015764 | 2.372 | 3.551 | 0.391 | 0.285 |
| Mtap7d3 | MAP7 domain containing 3 | NM_177293 | 2.315 | 3.907 | 0.301 | 0.104 |
| LOC105246057 | igE-binding protein-like | XR_875858 | 1.792 | 2.128 | 0.341 | 0.235 |
| Piezo2 | piezo-type mechanosensitive ion channel component 2 | NM_001039485 | 1.782 | 3.225 | 0.448 | 0.297 |
| Vmn2r48 | vomeronasal 2, receptor 48 | NM_001105152 | 1.588 | 3.253 | 0.243 | 0.101 |
| Tfcp2l1 | transcription factor CP2-like 1 | NM_023755 | 1.476 | 2.217 | 0.478 | 0.323 |
| Vmn2r35 | vomeronasal 2, receptor 35 | NM_001105067 | 1.296 | 3.750 | 0.253 | 0.091 |
| Fam170b | family with sequence similarity 170, member B | NM_001164485 | 0.386 | 2.907 | 0.325 | 0.036 |
| 2700022O18Rik | RIKEN cDNA 2700022O18 gene | AK012274 | 6.047 | 1.957 | 0.420 | 0.204 |
| Rhbdd1 | rhomboid domain containing 1 | NM_029777 | 10.902 | 1.952 | 0.423 | 0.218 |
| Myb | myeloblastosis oncogene | NM_001198914 | 8.191 | 1.952 | 0.100 | 0.352 |
| Dach2 | dachshund 2 (Drosophila) | NM_033605 | 2.794 | 1.923 | 0.225 | 0.405 |
| Rfesd | Rieske (Fe-S) domain containing | NM_178916 | 6.042 | 1.921 | 0.430 | 0.191 |
| 4930406D14Rik | RIKEN cDNA 4930406D14 gene | AK086320 | 3.892 | 1.920 | 0.479 | 0.268 |
| Wdr95 | WD40 repeat domain 95 | NM_029440 | 3.915 | 1.913 | 0.185 | 0.171 |
| Frmd4b | FERM domain containing 4B | NM_145148 | 2.136 | 1.907 | 0.457 | 0.276 |
| 6330522J23Rik | RIKEN cDNA 6330522J23 gene | AK020094 | 5.513 | 1.903 | 0.423 | 0.382 |
| Klkb1 | kallikrein B, plasma 1 | NM_008455 | 5.631 | 1.875 | 0.238 | 0.343 |
| Srek1 | splicing regulatory glutamine/lysine-rich protein 1 | AK164603 | 6.291 | 1.833 | 0.163 | 0.401 |
| Eomes | eomesodermin homolog (Xenopus laevis) | NM_010136 | 19.424 | 1.827 | 0.062 | 0.406 |
| Duoxa1 | dual oxidase maturation factor 1 | NM_001305262 | 1.536 | 1.821 | 0.457 | 0.225 |
| Marveld3 | MARVEL (membrane-associating) domain containing 3 | NM_212447 | 5.523 | 1.793 | 0.282 | 0.463 |
| Cpne1 | copine I | NM_170588 | 3.946 | 1.767 | 0.448 | 0.237 |
| C130023C23Rik | Riken cDNA C130023C23 gene | AK047948 | 5.794 | 1.766 | 0.425 | 0.299 |
| Spata18 | spermatogenesis associated 18 | NM_178387 | 5.827 | 1.741 | 0.359 | 0.403 |
| Thbs1 | thrombospondin 1 | NM_011580 | 3.000 | 1.736 | 0.305 | 0.471 |
| Fbxo24 | F-box protein 24 | NM_027708 | 7.214 | 1.729 | 0.373 | 0.340 |
| Adamtsl1 | ADAMTS-like 1 | AK020115 | 5.399 | 1.718 | 0.473 | 0.386 |
| Speer8-ps1 | spermatogenesis associated glutamate (E)-rich protein 8, pseudogene 1 | NR_001584 | 5.764 | 1.718 | 0.484 | 0.390 |
| Ptx4 | pentraxin 4 | NM_026747 | 1.737 | 1.707 | 0.272 | 0.277 |
| Ceacam2 | carcinoembryonic antigen-related cell adhesion molecule 2 | NM_001113368 | 0.639 | 1.704 | 0.491 | 0.196 |
| Cfap43 | cilia and flagella associated protein 43 | AK015217 | 13.472 | 1.691 | 0.098 | 0.377 |
| Gm5067 | predicted gene 5067 | AK031269 | 8.876 | 1.681 | 0.393 | 0.484 |
| Gm4907 | predicted gene 4907 | NM_001034864 | 1.866 | 1.679 | 0.438 | 0.373 |
| 5730407M17Rik | RIKEN cDNA 5730407M17 gene | AK017517 | 1.571 | 1.665 | 0.474 | 0.218 |
| Fcrlb | Fc receptor-like B | NM_001029984 | 6.512 | 1.640 | 0.472 | 0.242 |
| Armc4 | armadillo repeat containing 4 | NM_001081393 | 6.321 | 1.640 | 0.087 | 0.444 |
| Glb1l | galactosidase, beta 1-like | NM_029010 | 4.565 | 1.637 | 0.474 | 0.345 |
| Sp8 | trans-acting transcription factor 8 | NM_177082 | 11.968 | 1.633 | 0.108 | 0.431 |
| Syt10 | synaptotagmin X | NM_018803 | 34.035 | 1.618 | 0.346 | 0.186 |
| Uhrf1 | ubiquitin-like, containing PHD and RING finger domains, 1 | NM_010931 | 2.063 | 1.605 | 0.465 | 0.328 |
| Rsph4a | radial spoke head 4 homolog A (Chlamydomonas) | NM_001162957 | 3.368 | 1.603 | 0.283 | 0.328 |
| 4833411C07Rik | RIKEN cDNA 4833411C07 gene | BC052524 | 1.625 | 1.600 | 0.480 | 0.452 |
| Magt1 | magnesium transporter 1 | NM_025952 | 2.620 | 1.594 | 0.463 | 0.455 |
| Fignl1 | fidgetin-like 1 | NM_001163359 | 3.991 | 1.591 | 0.445 | 0.283 |
| Gnat2 | guanine nucleotide binding protein, alpha transducing 2 | NM_008141 | 8.322 | 1.589 | 0.392 | 0.331 |
| Reck | reversion-inducing-cysteine-rich protein with kazal motifs | NM_016678 | 6.018 | 1.576 | 0.444 | 0.302 |
| Car9 | carbonic anhydrase 9 | NM_139305 | 4.241 | 1.568 | 0.122 | 0.490 |
| Exd1 | exonuclease 3'-5' domain containing 1 | NM_172857 | 4.545 | 1.556 | 0.293 | 0.440 |
| Hacd4 | 3-hydroxyacyl-CoA dehydratase 4 | NM_025760 | 1.198 | 1.541 | 0.494 | 0.444 |
| LOC102634042 | uncharacterized LOC102634042 | XR_865201 | 1.366 | 1.531 | 0.463 | 0.079 |
| Nkapl | NFKB activating protein-like | NM_025719 | 1.704 | 1.528 | 0.435 | 0.378 |
| Mirg | miRNA containing gene | EU616813 | 15.534 | 1.508 | 0.339 | 0.305 |
| Lrguk | leucine-rich repeats and guanylate kinase domain containing | AK045903 | 5.401 | 1.483 | 0.303 | 0.301 |
| Cfap53 | cilia and flagella associated protein 53 | NM_028948 | 5.426 | 1.482 | 0.170 | 0.459 |
| Fam216b | family with sequence similarity 216, member B | NM_177629 | 5.893 | 1.478 | 0.157 | 0.429 |
| Myo1d | myosin ID | NM_177390 | 4.563 | 1.455 | 0.458 | 0.453 |
| Gm5606 | predicted gene 5606 | AK053768 | 3.814 | 1.454 | 0.387 | 0.301 |
| Ttc25 | tetratricopeptide repeat domain 25 | NM_028918 | 4.723 | 1.444 | 0.306 | 0.475 |
| Gm2590 | predicted gene 2590 | AK142040 | 5.062 | 1.443 | 0.391 | 0.489 |
| Dmkn | dermokine | NM_001166173 | 5.912 | 1.436 | 0.072 | 0.484 |
| Cdkn2b | cyclin-dependent kinase inhibitor 2B (p15, inhibits CDK4) | NM_007670 | 1.890 | 1.435 | 0.394 | 0.393 |
| Anxa8 | annexin A8 | NM_013473 | 3.337 | 1.428 | 0.224 | 0.398 |
| Gm4890 | predicted gene 4890 | NR_045822 | 2.760 | 1.402 | 0.306 | 0.468 |
| Ano2 | anoctamin 2 | NM_153589 | 5.020 | 1.389 | 0.245 | 0.428 |
| Sned1 | sushi, nidogen and EGF-like domains 1 | NM_172463 | 4.281 | 1.382 | 0.431 | 0.476 |
| Esr1 | estrogen receptor 1 (alpha) | NM_007956 | 1.088 | 1.377 | 0.302 | 0.375 |
| Dennd4a | DENN/MADD domain containing 4A | XM_011242654 | 1.402 | 1.373 | 0.468 | 0.379 |
| Tcp11 | t-complex protein 11 | NM_013687 | 6.883 | 1.366 | 0.259 | 0.409 |
| Sept10 | septin 10 | NM_001024910 | 2.203 | 1.364 | 0.430 | 0.486 |
| Gm13298 | predicted gene 13298 | NM_001085530 | 1.967 | 1.364 | 0.262 | 0.414 |
| Maats1 | MYCBP-associated, testis expressed 1 | NM_001081025 | 7.957 | 1.363 | 0.081 | 0.422 |
| Gm5607 | predicted gene 5607 | NR_027975 | 4.396 | 1.357 | 0.373 | 0.453 |
| 4732419C18Rik | RIKEN cDNA 4732419C18 gene | XR_863724 | 4.255 | 1.355 | 0.269 | 0.486 |
| Trim68 | tripartite motif-containing 68 | NM_198012 | 1.956 | 1.338 | 0.492 | 0.421 |
| Cpped1 | calcineurin-like phosphoesterase domain containing 1 | NM_146067 | 3.129 | 1.332 | 0.423 | 0.128 |
| Car8 | carbonic anhydrase 8 | NM_007592 | 0.789 | 1.328 | 0.184 | 0.475 |
| Cobll1 | Cobl-like 1 | NM_177025 | 3.420 | 1.327 | 0.470 | 0.456 |
| Ggt6 | gamma-glutamyltransferase 6 | NM_027819 | 2.630 | 1.323 | 0.490 | 0.418 |
| Scin | scinderin | NM_009132 | 1.074 | 1.318 | 0.485 | 0.287 |
| Ccdc151 | coiled-coil domain containing 151 | NM_001163787 | 1.348 | 1.310 | 0.415 | 0.412 |
| Arhgap6 | Rho GTPase activating protein 6 | NM_009707 | 7.322 | 1.309 | 0.185 | 0.486 |
| Sp9 | trans-acting transcription factor 9 | NM_001005343 | 5.189 | 1.305 | 0.262 | 0.414 |
| Kiss1 | KiSS-1 metastasis-suppressor | NM_178260 | 1.969 | 1.303 | 0.413 | 0.483 |
| 1700101I19Rik | RIKEN cDNA 1700101I19 gene | AK007108 | 6.753 | 1.286 | 0.477 | 0.317 |
| Myh6 | myosin, heavy polypeptide 6, cardiac muscle, alpha | NM_001164171 | 2.219 | 1.286 | 0.453 | 0.483 |
| Lrrc36 | leucine rich repeat containing 36 | NM_001170788 | 8.390 | 1.283 | 0.136 | 0.456 |
| Eppk1 | epiplakin 1 | BC026387 | 6.846 | 1.274 | 0.123 | 0.371 |
| Tcte1 | t-complex-associated testis expressed 1 | NM_013688 | 2.534 | 1.272 | 0.429 | 0.482 |
| Cenpe | centromere protein E | NM_173762 | 0.712 | 1.266 | 0.496 | 0.307 |
| Vax1 | ventral anterior homeobox 1 | NM_009501 | 4.056 | 1.255 | 0.195 | 0.459 |
| C230073G13Rik | RIKEN cDNA C230073G13 gene | AK082636 | 4.216 | 1.247 | 0.185 | 0.435 |
| Usp51 | ubiquitin specific protease 51 | NM_001137547 | 1.747 | 1.239 | 0.358 | 0.380 |
| C230030N03Rik | RIKEN cDNA C230030N03 gene | AK082264 | 1.822 | 1.208 | 0.399 | 0.472 |
| Esyt3 | extended synaptotagmin-like protein 3 | NM_177775 | 2.932 | 1.206 | 0.311 | 0.436 |
| Slc9a3 | solute carrier family 9 (sodium/hydrogen exchanger), member 3 | XM_006517022 | 1.347 | 1.205 | 0.446 | 0.455 |
| 5830443J22Rik | RIKEN cDNA 5830443J22 gene | AK017987 | 2.374 | 1.204 | 0.494 | 0.439 |
| Sctr | secretin receptor | NM_001012322 | 1.905 | 1.200 | 0.272 | 0.440 |
| Lrp2 | low density lipoprotein receptor-related protein 2 | NM_001081088 | 2.305 | 1.189 | 0.178 | 0.376 |
| Iqca | IQ motif containing with AAA domain | NM_029122 | 4.536 | 1.185 | 0.106 | 0.472 |
| Gimap4 | GTPase, IMAP family member 4 | NM_174990 | 6.190 | 1.184 | 0.474 | 0.373 |
| AW551984 | expressed sequence AW551984 | NM_178737 | 3.437 | 1.181 | 0.121 | 0.464 |
| Stom | stomatin | NM_013515 | 0.755 | 1.175 | 0.414 | 0.240 |
| Tex26 | testis expressed 26 | NM_029464 | 4.201 | 1.165 | 0.360 | 0.481 |
| Dock10 | dedicator of cytokinesis 10 | NM_001285927 | 1.476 | 1.140 | 0.410 | 0.348 |
| 4931406E20Rik | RIKEN cDNA 4931406E20 gene | AK163891 | 4.706 | 1.139 | 0.394 | 0.481 |
| Palmd | palmdelphin | AK136939 | 1.039 | 1.120 | 0.483 | 0.329 |
| Ldoc1 | leucine zipper, down-regulated in cancer 1 | NM_001018087 | 1.262 | 1.113 | 0.491 | 0.486 |
| Lrba | LPS-responsive beige-like anchor | NM_030695 | 1.226 | 1.099 | 0.448 | 0.443 |
| Slc39a9 | solute carrier family 39 (zinc transporter), member 9 | NM_026244 | 1.726 | 1.097 | 0.499 | 0.284 |
| Aldh3b2 | aldehyde dehydrogenase 3 family, member B2 | NM_001177438 | 3.460 | 1.080 | 0.183 | 0.457 |
| Ostf1 | osteoclast stimulating factor 1 | NM_017375 | 1.909 | 1.079 | 0.499 | 0.473 |
| Itgb1 | integrin beta 1 (fibronectin receptor beta) | NM_010578 | 1.537 | 1.073 | 0.476 | 0.453 |
| Pggt1b | protein geranylgeranyltransferase type I, beta subunit | NM_172627 | 1.662 | 1.068 | 0.490 | 0.473 |
| Agbl2 | ATP/GTP binding protein-like 2 | NM_178755 | 2.284 | 1.065 | 0.384 | 0.443 |
| Atp6v1c2 | ATPase, H+ transporting, lysosomal V1 subunit C2 | NM_133699 | 2.293 | 1.062 | 0.311 | 0.455 |
| Dclre1c | DNA cross-link repair 1C, PSO2 homolog (S. cerevisiae) | NM_175683 | 0.661 | 1.058 | 0.462 | 0.482 |
| A330070K13Rik | RIKEN cDNA A330070K13 gene | NM_198665 | 0.821 | 1.047 | 0.450 | 0.412 |
| Oxtr | oxytocin receptor | NM_001081147 | 0.956 | 1.033 | 0.456 | 0.335 |
| Rasl12 | RAS-like, family 12 | NM_001033158 | 0.941 | 1.029 | 0.468 | 0.481 |
| Dlx2 | distal-less homeobox 2 | NM_010054 | 2.691 | 1.028 | 0.335 | 0.378 |
| Hist1h2bc | histone cluster 1, H2bc | NM_023422 | 1.217 | 1.027 | 0.395 | 0.477 |
| Vwa3a | von Willebrand factor A domain containing 3A | NM_177697 | 3.529 | 1.021 | 0.229 | 0.464 |
| Cpne3 | copine III | NM_027769 | 2.225 | 1.012 | 0.460 | 0.417 |
| Ccdc68 | coiled-coil domain containing 68 | NM_201362 | 1.700 | 1.007 | 0.358 | 0.447 |
| N4bp2 | NEDD4 binding protein 2 | NM_001024917 | 1.389 | 1.001 | 0.484 | 0.484 |
| Gm5089 | predicted gene 5089 | AK047660 | 2.168 | 1.001 | 0.358 | 0.483 |
| Cep41 | centrosomal protein 41 | NM_031998 | 1.737 | 1.000 | 0.381 | 0.488 |
| Gm14484 | predicted gene 14484 | NM_001025260 | 2.853 | 0.999 | 0.377 | 0.443 |
| Zfp780b | zinc finger protein 780B | NM_001081021 | 0.317 | 0.998 | 0.336 | 0.463 |
| Gna14 | guanine nucleotide binding protein, alpha 14 | NM_008137 | 1.128 | 0.996 | 0.432 | 0.443 |
| Lman1 | lectin, mannose-binding, 1 | NM_001172062 | 1.337 | 0.995 | 0.469 | 0.487 |
| Tcerg1l | transcription elongation regulator 1-like | NM_183289 | 1.766 | 0.994 | 0.274 | 0.465 |
| Fndc1 | fibronectin type III domain containing 1 | NM_001081416 | 0.924 | 0.992 | 0.489 | 0.439 |
| Amd2 | S-adenosylmethionine decarboxylase 2 | NM_007444 | 1.632 | 0.992 | 0.497 | 0.472 |
| Gmnc | geminin coiled-coil domain containing | NM_001285916 | 1.152 | 0.984 | 0.341 | 0.288 |
| 4922502B01Rik | RIKEN cDNA 4922502B01 gene | AK015021 | 4.953 | 0.973 | 0.408 | 0.411 |
| Mapk1 | mitogen-activated protein kinase 1 | NM_001038663 | 0.676 | 0.970 | 0.231 | 0.490 |
| Car12 | carbonic anyhydrase 12 | NM_178396 | 1.316 | 0.966 | 0.362 | 0.461 |
| Slit2 | slit homolog 2 (Drosophila) | NM_001291227 | 1.428 | 0.959 | 0.351 | 0.304 |
| Masp2 | mannan-binding lectin serine peptidase 2 | NM_010767 | 3.900 | 0.951 | 0.290 | 0.452 |
| Cpne2 | copine II | NM_153507 | 1.228 | 0.945 | 0.293 | 0.499 |
| Col11a1 | collagen, type XI, alpha 1 | NM_007729 | 8.904 | 0.943 | 0.422 | 0.413 |
| Prokr2 | prokineticin receptor 2 | NM_144944 | 1.075 | 0.929 | 0.423 | 0.408 |
| Mettl20 | methyltransferase like 20 | AK077292 | 1.288 | 0.914 | 0.425 | 0.385 |
| Igfbpl1 | insulin-like growth factor binding protein-like 1 | NM_018741 | 11.818 | 0.908 | 0.134 | 0.285 |
| Trp63 | transformation related protein 63 | NM_011641 | 1.040 | 0.890 | 0.481 | 0.408 |
| Ptgr1 | prostaglandin reductase 1 | NM_025968 | 0.740 | 0.887 | 0.498 | 0.453 |
| Gaa | glucosidase, alpha, acid | NM_008064 | 0.956 | 0.874 | 0.414 | 0.287 |
| Mdm1 | transformed mouse 3T3 cell double minute 1 | NM_001162904 | 1.733 | 0.871 | 0.430 | 0.424 |
| Adamts18 | a disintegrin-like and metallopeptidase (reprolysin type) with thrombospondin type 1 motif, 18 | NM_172466 | 1.400 | 0.865 | 0.453 | 0.454 |
| Nnmt | nicotinamide N-methyltransferase | NM_010924 | 0.766 | 0.855 | 0.390 | 0.477 |
| Spink8 | serine peptidase inhibitor, Kazal type 8 | NM_183136 | 0.553 | 0.840 | 0.394 | 0.426 |
| Lamb3 | laminin, beta 3 | NM_008484 | 2.044 | 0.838 | 0.463 | 0.463 |
| Plet1 | placenta expressed transcript 1 | NM_029639 | 1.551 | 0.822 | 0.329 | 0.300 |
| Gpr101 | G protein-coupled receptor 101 | NM_001033360 | 1.565 | 0.801 | 0.335 | 0.406 |
| Tox4 | TOX high mobility group box family member 4 | NM_023434 | 2.352 | 0.697 | 0.455 | 0.250 |
| Tshz2 | teashirt zinc finger family member 2 | NM_080455 | 0.558 | 0.250 | 9.865 | 3.991 |
| Bglap3 | bone gamma-carboxyglutamate protein 3 | NM_001305448 | 1.176 | 0.407 | 4.453 | 2.058 |
| Ctgf | connective tissue growth factor | NM_010217 | 0.423 | 0.666 | 4.330 | 2.067 |
| Epha8 | Eph receptor A8 | NM_007939 | 0.843 | 0.742 | 2.144 | 2.135 |
| Amn | amnionless | NM_033603 | 0.724 | 0.752 | 4.681 | 2.005 |
| Ramp3 | receptor (calcitonin) activity modifying protein 3 | AF209907 | 0.478 | 1.016 | 2.953 | 2.496 |
| Fmod | fibromodulin | NM_021355 | 0.736 | 1.024 | 2.669 | 2.016 |
| H2-Q1 | histocompatibility 2, Q region locus 1 | NM_010390 | 0.241 | 1.079 | 7.193 | 2.872 |
| Wnt6 | wingless-type MMTV integration site family, member 6 | NM_009526 | 1.087 | 1.145 | 2.782 | 2.474 |

**Figure S1.** **Protein-Protein interaction network analysis of selected genes in both young and middle-aged hippocampi following chronic RF-EMF.** Extended protein-protein interaction (PPI) network constructed by STRING database version 11.0 and visualized by Cytoscape version 3.7.2. The sub‑network reconstructed with the DE genes and their first neighbor genes.

**Figure S2.** **Changes in the levels of neurogenesis-related genes in the mouse hippocampus after chronic RF-EMF.** The bar graphs show the levels of mRNA encoding Cxcl5, Eomes, Vax1, Cpne1, Fmod, Gnat2, Itgb1, Mapk1, Pdrm12, Slit2, Dlx2, Duoxa1, and Sall3 in the hippocampi of 10M and 20M mice. All data are reported as mean ± SEM (n = 5 per group).
